# Association of Social Distancing, Population Density, and Temperature With the Instantaneous Reproduction Number of SARS-CoV-2 in Counties Across the United States

**DOI:** 10.1001/jamanetworkopen.2020.16099

**Published:** 2020-07-23

**Authors:** David Rubin, Jing Huang, Brian T. Fisher, Antonio Gasparrini, Vicky Tam, Lihai Song, Xi Wang, Jason Kaufman, Kate Fitzpatrick, Arushi Jain, Heather Griffis, Koby Crammer, Jeffrey Morris, Gregory Tasian

**Affiliations:** 1 Department of Pediatrics, Children’s Hospital of Philadelphia, Philadelphia, Pennsylvania; 2 Department of Pediatrics, Perelman School of Medicine at the University of Pennsylvania, Philadelphia; 3 PolicyLab, Children’s Hospital of Philadelphia, Philadelphia, Pennsylvania; 4 Department of Biostatistics, Epidemiology, and Informatics, Perelman School of Medicine at the University of Pennsylvania, Philadelphia; 5 Division of Infectious Disease, Perelman School of Medicine at the University of Pennsylvania, Philadelphia; 6 Center for Pediatric Clinical Effectiveness, Children’s Hospital of Philadelphia, Philadelphia, Pennsylvania; 7 Department of Public Health Environments and Society, London School of Hygiene and Tropical Medicine, London, United Kingdom; 8 Centre for Statistical Methodology, London School of Hygiene and Tropical Medicine, London, United Kingdom; 9 Centre on Climate Change and Planetary Health, London School of Hygiene and Tropical Medicine, London, United Kingdom; 10 Data Science and Biostatistics Unit, Department of Biomedical and Health Informatics, Children’s Hospital of Philadelphia, Philadelphia, Pennsylvania; 11 Perelman School of Medicine at the University of Pennsylvania, Philadelphia; 12 Division of Urology, Department of Surgery, Children’s Hospital of Philadelphia, Philadelphia, Pennsylvania; 13 Department of Electrical Engineering, The Technion, Haifa, Israel

## Abstract

**Importance:**

Local variation in the transmission of severe acute respiratory syndrome coronavirus 2 (SARS-CoV-2) across the United States has not been well studied.

**Objective:**

To examine the association of county-level factors with variation in the SARS-CoV-2 reproduction number over time.

**Design, Setting, and Participants:**

This cohort study included 211 counties, representing state capitals and cities with at least 100 000 residents and including 178 892 208 US residents, in 46 states and the District of Columbia between February 25, 2020, and April 23, 2020.

**Exposures:**

Social distancing, measured by percentage change in visits to nonessential businesses; population density; and daily wet-bulb temperatures.

**Main Outcomes and Measures:**

Instantaneous reproduction number (R_t_), or cases generated by each incident case at a given time, estimated from daily case incidence data.

**Results:**

The 211 counties contained 178 892 208 of 326 289 971 US residents (54.8%). Median (interquartile range) population density was 1022.7 (471.2-1846.0) people per square mile. The mean (SD) peak reduction in visits to nonessential business between April 6 and April 19, as the country was sheltering in place, was 68.7% (7.9%). Median (interquartile range) daily wet-bulb temperatures were 7.5 (3.8-12.8) °C. Median (interquartile range) case incidence and fatality rates per 100 000 people were approximately 10 times higher for the top decile of densely populated counties (1185.2 [313.2-1891.2] cases; 43.7 [10.4-106.7] deaths) than for counties in the lowest density quartile (121.4 [87.8-175.4] cases; 4.2 [1.9-8.0] deaths). Mean (SD) R_t_ in the first 2 weeks was 5.7 (2.5) in the top decile compared with 3.1 (1.2) in the lowest quartile. In multivariable analysis, a 50% decrease in visits to nonessential businesses was associated with a 45% decrease in R_t_ (95% CI, 43%-49%). From a relative R_t_ at 0 °C of 2.13 (95% CI, 1.89-2.40), relative R_t_ decreased to a minimum as temperatures warmed to 11 °C, increased between 11 and 20 °C (1.61; 95% CI, 1.42-1.84) and then declined again at temperatures greater than 20 °C. With a 70% reduction in visits to nonessential business, 202 counties (95.7%) were estimated to fall below a threshold R_t_ of 1.0, including 17 of 21 counties (81.0%) in the top density decile and 52 of 53 counties (98.1%) in the lowest density quartile.^2^

**Conclusions and Relevance:**

In this cohort study, social distancing, lower population density, and temperate weather were associated with a decreased R_t_ for SARS-CoV-2 in counties across the United States. These associations could inform selective public policy planning in communities during the coronavirus disease 2019 pandemic.

## Key Points

**Question:**

How is the instantaneous reproduction number of severe acute respiratory syndrome coronavirus 2 (SARS-CoV-2) associated with social distancing, wet-bulb temperature, and population density in counties across the United States?

**Findings:**

In this cohort study of 211 counties in 46 states, social distancing, temperate weather, and lower population density were associated with a decrease in the instantaneous reproduction number of SARS-CoV-2. Of these county-specific factors, social distancing appeared to have the most substantial association with a reduction in SARS-CoV-2 transmission.

**Meaning:**

In this study, the instantaneous reproduction number of SARS-CoV-2 varied substantially among counties; the associations between the reproduction number and county-specific factors could inform policies to reduce SARS-CoV-2 transmission in selective and heterogeneous communities.

## Teaser

This cohort study examines the association of county-level factors with variation in the instantaneous reproduction number of severe acute respiratory syndrome coronavirus 2 (SARS-CoV-2) in the United States.

## Introduction

Coronavirus disease 2019 (COVID-19) is the result of the novel severe acute respiratory syndrome coronavirus 2 (SARS-CoV-2). This virus has caused a pandemic resulting in more than 3.2 million cases of COVID-19 by May 1, 2020, with more than 236 000 deaths worldwide. By the same date, there were more than 1 million individuals with COVID-19 in the United States, resulting in more than 64 000 deaths. The rapid evolution of this pandemic led to the widespread implementation of social distancing measures across most of the United States and the world.

The transmissibility of SARS-CoV-2, like other viral pathogens, is estimated by the reproduction number (R). An infectious pathogen’s R value represents the number of people that will be infected by an individual who has the infection. An R value that exceeds 1 will result in increasing numbers of incident cases as each individual with the infection transmits it to more than 1 other individual. When the R value is below 1, the transmission of that pathogen will eventually cease as each patient will transmit infection to less than 1 person. Therefore, the R value is an important measure to estimate when attempting to predict the evolution of an outbreak. It is often assumed that R is constant for each pathogen; however, R most certainly varies by location and by time, which is referred to as the instantaneous R (R_t_).^1,2,3^ At the individual level, variation in R_t_ is likely dependent on being in environments where exposure risk is high or of intense duration, such as among high-exposure workers in health care or mass transit settings or for families in densely crowded living conditions. At the community level, variation in R_t_ may also include population density (as a proxy for increased likelihood of crowded conditions), temperature and/or humidity (given their effects on viral propagation), policies such as social distancing, and the number of susceptible individuals.

Models that rely on fixed assumptions for R_t_ are unlikely to capture local heterogeneity in transmission. Our objective was to examine how time-varying changes in social distancing and weather within counties of different population densities might be associated with changing R_t_ values across counties in the United States. Understanding how these time-varying factors might influence the R_t_ of SARS-CoV-2 could allow policy makers to implement targeted interventions to decrease R_t_ in heterogeneous communities.

## Methods

Using publicly deidentified data, this study was determined to be exempt from institutional review board review^4^ and informed consent. This study followed the Strengthening the Reporting of Observational Studies in Epidemiology (STROBE) reporting guideline for cohort studies.^5^

### Setting and Participants

We selected 211 counties, representing 178 892 208 of 326 289 971 US residents (54.8%), based on the following characteristics: had at least 1 case of COVID-19 as of February 25, 2020, and either contained at least 1 city with population exceeding 100 000 residents or the state capital. For states with no counties containing a city with 100 000 persons, the most populated county in that state was selected. We excluded counties with average daily case rates of less than 5 and counties with fewer than 3 days with daily case rates of more than 5 during the analysis period of February 25 to April 23, 2020. We considered time-0 for each county to be the date on which they achieved the minimum threshold of disease activity. A total of 211 counties, representing 46 states and the District of Columbia, met these criteria.

### Outcome

The outcome was the estimated R_t_ of SARS-CoV-2 in each county. Daily incident case counts of COVID-19 aggregated at the county level were obtained from the *New York Times*.^6^

### Exposures

There were 3 a priori exposure variables: social distancing practice, population density, and daily mean wet-bulb temperatures. Social distancing was measured using a data set of daily cellular telephone movement, provided by Unacast, that allows comparison of the association of social distancing policies with individuals’ movement within a county.^7,8^ Based on an a priori assumption and confirmed by preliminary analyses that included cellular telephone measurement of overall distance traveled, we used a social distancing variable that measured percentage change in visits to nonessential businesses (eg, restaurants, hair salons) within each county compared with visits in a 4-week baseline period between February 10 and March 8, 2020. We used a rolling average of the percentage of visits 3 to 14 days before time-0, based on the lag observed between change in social distancing and mean R_t_ estimates across the counties (eFigure 1 in the Supplement) and on an incubation period of at least 3 days.^9^

Population density of each county was obtained from US Census data and is expressed as number of people per square mile. Log transformation was performed to achieve a normal distribution because of substantial skewedness in density for the largest cities.

It has been demonstrated that humidity and temperature both play a role in the seasonality of influenza; viral transmission is most efficient at lower humidity and temperature. It is proposed that colder, drier air damages respiratory mucosa and thickens secretions, impairing the protective capacity of mucociliary clearance, and that higher humidity physically limits the distance respiratory droplets can travel.^10,11^ For this reason, the primary weather variable we used was wet-bulb temperature, a metric that captures the complex thermodynamic relationship of temperature and humidity, has been shown to predict human health events with more precision than temperature and humidity separately, and avoids the associated problem of collinearity.^12,13^ Wet-bulb temperatures were obtained from the National Oceanic and Atmospheric Administration Local Climatological Data.

### Covariates

County-level covariates that may confound the association between the exposures of interest and R_t_ were considered and included demographic factors (eg, age distribution, insurance status, and socioeconomic status) and health-related factors associated with COVID-19 severity (eg, proportion of individuals with hypertension, obesity, or diabetes and proportion of individuals who smoke).^14^ Demographic and health characteristics were abstracted from the US Census, American Community Survey, Behavioral Risk Factor Surveillance System, Esri Business Analyst, and Multi-Resolution Land Characteristics Consortium.^15,16,17,18^

From 70 covariates, we examined the correlation between each pair of factors and calculated the variance inflation factor to quantify multicollinearity among variables (eFigure 2 in the Supplement). Among highly correlated variables, we chose covariates based on their potential association with viral transmission and/or the probability of an individual with infection becoming symptomatic and obtaining a diagnostic test. The final covariates included proportion of residents older than 65 years, with incomes less than 200% of the poverty level, and with diabetes. Variables for obesity, smoking, and uninsured population were removed because of collinearity with other variables.

### Statistical Analysis

Our analysis followed a 2-step procedure. First, we calculated the R_t_ for SARS-CoV-2 using the method of Cori et al^1^ with a moving average window of 3 days. This method has been applied in the dynamic estimation of R_t_ from Wuhan, China.^19^ The generation time of SARS-CoV-2 was assumed to follow a γ distribution, with mean (SD) of 7.5 (3.4) days according to a previous epidemiological survey of the first 425 cases in Wuhan, China.^9^ In the early days of a county’s outbreak, when the ratio of cases to tests was unstable, kernel smoothing with a box kernel and bandwidth of 7 days was performed to account for the likelihood that cases from prior days would accumulate when testing capacity increased.^20,21^ We stopped smoothing 1 day after the county’s ratio of daily cases to tests first fell in the interval of 5% to 90% or after March 20, 2020, whichever came first, to avoid overprocessing the data.

Next, we fit a hierarchical linear mixed-effects model with random intercepts for each county and metropolitan area to evaluate the association between exposures and R_t_ after a log transformation, adjusting for covariates. Population density was standardized after log transformation because of the highly skewed distribution. Temperature associations were estimated using a distributed lag nonlinear model, which considers bidimensional exposure-lag response associations between wet-bulb temperature and log(R_t_).^22,23,24^ We considered a lag period of 4 days to 14 days before case identification to reflect the incubation period of SARS-CoV-2 and to reduce bias introduced by daily weather affecting an individual’s decision to seek a test. The final cross-basis term included natural cubic splines defined by 3 internal knots at the 10th, 75th, and 90th percentile of temperature ranges observed during the period, corresponding to 1 °C, 13 °C, and 19 °C, and 2 knots in the lag dimension at 7 and 11 days. The number and placement of spline knots were based on an a priori assumption of a relatively simple association between temperature and SARS-CoV-2 transmission and on minimization of the Akaike information criterion. The temperature knots permitted flexibility in the model nonlinearity at higher and lower temperatures, within the moderate range used in this study.^25^ We included an interaction term between population density and temperature, assuming that temperatures would influence transmissibility differently in densely populated counties.^26^ The relative change in R_t_ was expressed as the cumulative exposure response relative to 11 °C. Interactions between population density and social distancing were included in the linear mixed-effects model, given the hypothesis that the association of social distancing with the R_t_ might be greater in highly dense areas. We also controlled for potential time effect using a cubic polynomial of days in outbreak. Statistical significance of the associations was determined using the maximum likelihood ratio test at the nominal level of *P* < .05. All tests were 2-tailed.

We ran 3 sensitivity analyses. First, the model was re-estimated every 2 weeks (a total of 3 times) during a period of 1 month, checking for stability in estimates of associations for primary covariates. Second, the model fit was evaluated by calculating in-sample *R*^2^ in a randomly selected 70% of counties over 100 replicates. Finally, to address concerns regarding potential bias owing to the exclusion of counties with later outbreaks or with less overall population density, we relaxed our inclusion criteria to permit counties with active outbreaks and a total population of at least 100 000 residents (as opposed to having cities with at least 100 000 residents), and we re-estimated the associations of social distancing and wet-bulb temperatures with R_t_ during the study period. Given concerns regarding limited representation of temperatures at the upper and lower ranges across counties of different population density, we did not include interaction terms between population density and temperature. Analyses were performed with R version 3.6.0 (R Project for Statistical Computing) using the EpiEstim and dlnm packages.^27^

## Results

**Figure 1.**
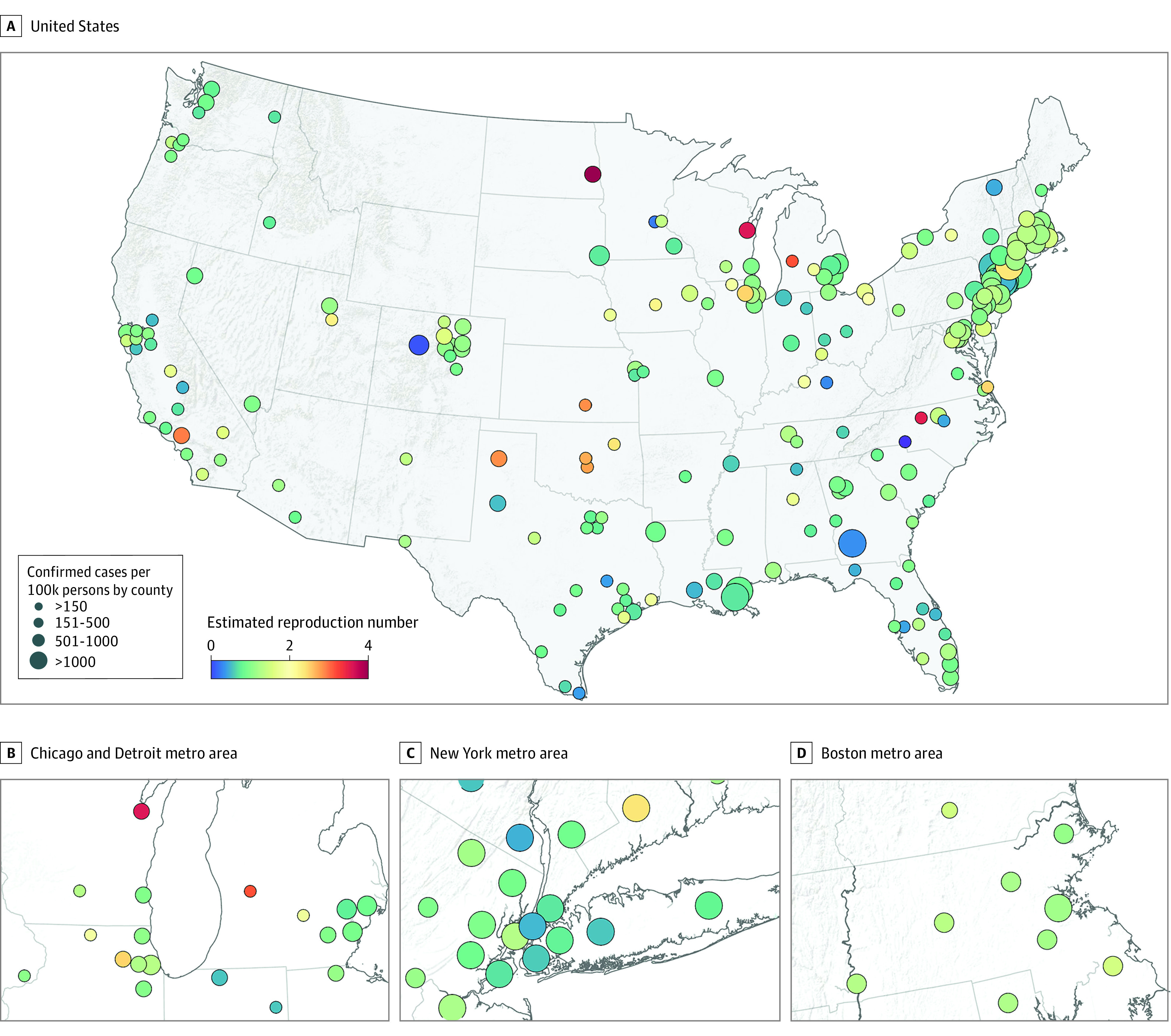
Location and Estimated Instantaneous Reproduction Number of Severe Acute Respiratory Syndrome Coronavirus 2 as of April 26, 2020, in 211 Counties in the United States

Geographic locations and characteristics of the 211 counties are demonstrated in Figure 1 and Table 1. The 211 counties contained 178 892 208 of 326 289 971 US residents (54.8%). County median (interquartile range [IQR]) population density was 1022.7 (471.2-1846.0) people per square mile, with skewing of the top decile of 21 counties to a median (IQR) of 8916.0 (5381.1-14 475.6) people per square mile. The mean (SD) reduction in visits by people to nonessential business by mid-April was 68.7% (7.9%). Median (IQR) daily wet-bulb temperatures were 7.5 (3.8-12.8) °C. The 21 counties in the top decile for population density had the highest median (IQR) incident case and fatality rate per 100 000 people (1185.2 [313.2-1891.2] cases; 43.7 [10.4-106.7]), nearly 10 times the estimates in the lowest quartile (121.4 [87.8-175.4] cases; 4.2 (1.9-8.0) deaths). Mean (SD) R in the first 2 weeks was 5.7 (2.5) in the top decile compared with 3.1 (1.2) in the lowest quartile. The mean (SD) change in visits to nonessential businesses from April 6 to April 19 was higher among counties in the top decile (−77.9% [7.9%]) compared with those in the lowest quartile (−66.3% [7.8%]). The top decile of counties also experienced colder median (IQR) temperatures during the analysis period compared with the counties in the lowest quartile (5.9 [3.4-8.4] °C vs 7.9 [3.1-12.7] °C).

**Table 1.** Population Characteristics in an Analysis of Instantaneous R for Severe Acute Respiratory Syndrome Coronavirus 2 by Population Density for 211 Counties Across the United States

Characteristic^a^	Population density					
	<25th Percentile (n = 53)	25th-50th Percentile (n = 53)	50th-75th Percentile (n = 53)	75th-90th Percentile (n = 31)	>90th Percentile (n = 21)	All (N = 211)
Population density, median (IQR), people per square mile	298.4 (164.2-376.1)	662.7 (539.1-826.8)	1368.7 (1211.4-1584.9)	2395.4 (2151.2-2860.6)	8916.0 (5381.8-14 475.6)	1022.7 (471.2-1846.0)
Hypertension, mean (SD), % of population	29.4 (5.3)	31.6 (3.9)	31.2 (3.1)	31.5 (3.8)	29.3 (3.5)	30.7 (4.2)
Diabetes, mean (SD), % of population	9.2 (2.3)	10.1 (1.8)	10.0 (1.3)	10.3 (1.5)	10.1 (1.5)	9.9 (1.8)
Regular smoking, mean (SD), % of population^b^	16.5 (2.4)	16.9 (2.3)	16.3 (2.6)	16.6 (2.7)	15.9 (2.5)	16.5 (2.5)
BMI >30, mean (SD), % of population	29.6 (4.5)	30.9 (3.5)	30.2 (3.5)	31.1 (3.8)	28.2 (4.1)	30.1 (3.9)
Age, median (IQR), % of population						
<18 y	23.9 (22.1-25.7)	23.4 (21.6-24.8)	22.3 (21.4-23.8)	23.1 (22.1-24.1)	20.9 (18.3-22.2)	22.7 (21.4-24.5)
18-34 y	24.5 (23.1-26.4)	23.7 (22.0-26.1)	23.3 (21.3-25.0)	24.4 (22.7-25.9)	28.3 (23.8-30.6)	24.1 (22.3-26.2)
35-64 y	37.1 (35.5-38.6)	38.1 (36.5-39.8)	39.5 (38.1-41.0)	38.9 (37.6-40.3)	38.8 (37.1-40.6)	38.4 (36.9-40.1)
≥65 y	13.8 (11.9-14.8)	13.5 (12.6-15.2)	14.7 (12.8-16.2)	13.5 (11.8-14.7)	13.2 (11.9-14.8)	13.8 (12.4-15.4)
Low income, mean (SD), % of population^c^	33.2 (9.8)	31.5 (8.6)	27.0 (8.8)	30.4 (8.5)	31.2 (10.2)	30.6 (9.3)
Uninsured, mean (SD), % of population	9.6 (4.7)	9.5 (5.4)	8.2 (4.1)	9.4 (4.4)	8.5 (3.1)	9.1 (4.6)
Change in visits to nonessential businesses, mean (SD), %^d^						
February 24 to March 8	–2.4 (7.0)	–2.5 (6.1)	–3.0 (5.7)	–3.1 (6.1)	–2.8 (6.3)	–2.7 (6.3)
April 6 to April 19	–66.3 (7.8)	–65.3 (7.3)	–69.2 (6.8)	–71.3 (5.8)	–77.9 (5.2)	–68.7 (7.9)
Daily wet-bulb temperature, median (IQR), °C^e^	7.9 (3.1-12.7)	8.2 (4.1-14.8)	8.2 (4.2-14.5)	7.8 (3.9-13.7)	5.9 (3.4-8.4)	7.5 (3.8-12.8)
R in the first 2 weeks, mean (SD)	3.1 (1.2)	3.1 (1.1)	3.6 (1.1)	4.0 (1.6)	5.7 (2.5)	3.6 (1.6)
Cases per 100 000 people on April 26, median (IQR), No.	121.4 (87.8-175.4)	126.7 (79.3-180.3)	196.0 (73.1-530.4)	206.6 (110.5-483.2)	1185.2 (313.2-1891.2)	154.7 (87.9-350.6)
Deaths per 100 000 people on April 26, median (IQR), No.	4.2 (1.9-8.0)	4.2 (2.3-7.5)	5.6 (2.3-28.6)	8.9 (4.1-21.9)	43.7 (10.4-106.7)	5.8 (2.5-16.3)

Abbreviations: BMI, body mass index (calculated as weight in kilograms divided by height in meters squared); IQR, interquartile range; R, reproduction number.

^a^ All characteristics were obtained from the American Community Survey (2018) except health data, which were obtained from the US Centers for Disease Control and Prevention Behavioral Risk Factor Surveillance System (2017); social distancing, which were obtained from Unacast (2020); and wet-bulb temperature, which were obtained from the National Oceanic and Atmospheric Administration (2020).

^b^ Regular smoking was defined as adult respondents who reported smoking at least 100 cigarettes in their life and currently smoking at least some days.

^c^ Low income was defined as incomes of less than 200% of the poverty level.

^d^ Visits to nonessential businesses obtained from Unacast, calculated as the change from mean nonessential business visits during matching days of the week before March 9, 2020.

^e^ Daily wet-bulb temperatures were calculated by averaging the hourly recordings from weather stations that contribute to the National Oceanic and Atmospheric Administration Local Climatological Data.

We estimated R_t_ over a total of 6588 county-days for the 211 counties; 17 outlier county days were removed because of an extremely low estimate of R_t_ at low case thresholds (ie, R_t_ < 0.05). The estimated R_t_ by county varied greatly, but it tended to be highest earlier the epidemic, reaching a peak R_t_ of 7.8 before declining later in the period (eFigure 1 in the Supplement). Adjusting for county-level covariates, social distancing, population density, and temperature were associated with R_t_ (Table 2). The estimated R_t_ in the context of a 50% decrease in visits to nonessential businesses was 54% (95% CI, 51%-57%; *P* < .001) of the R_t_ in the setting of normal visit intensity, corresponding to a 46% decrease in the overall R_t_. Compared with counties in the bottom quartile of population density, the 21 counties in the top decile of density had a 15% increase (95% CI, 9%-22%; *P* < .001) in relative R_t_.

**Table 2.** Ratios of R_t_ for Social Distancing and Population Density in 211 United States Counties Between February 25 and April 23, 2020

Variable	R_t_ ratio estimates with 95% CIs^a^			*P* value
Visits to nonessential businesses, compared with no change^b^	Reduce 25%	Reduce 50%	Reduce 75%	NA
	0.73 (0.71-0.75)	0.54 (0.51-0.57)	0.40 (0.36-0.43)	<.001
Population density, compared with 25th percentile^c^	50th percentile	75th percentile	90th percentile	NA
	1.05 (1.03-1.07)	1.09 (1.05-1.14)	1.15 (1.09-1.22)	<.001
Wet-bulb temperature	0 °C	5 °C	20 °C	NA
	2.13 (1.89-2.40)	1.38 (1.27- 1.50)	1.61 (1.41-1.84)	<.001

Abbreviations: NA, not applicable; R_t_, instantaneous reproduction number.

^a^ Estimates and variation obtained through mixed-effects linear models using a log transformed R_t_, log population density, and distributed lag nonlinear models to estimate temperature effects. Postestimation was performed to convert variables into meaningful units of change. Ratios of R_t_ are compared with the reference groups, adjusting for proportion of residents older than 65 years, with incomes less than 200% of the poverty level, and with diabetes. Marginal *R*^2^ was 0.50; conditional *R*^2^ was 0.61.

^b^ Visits to nonessential businesses obtained from Unacast. The referent value was the average visits to nonessential business before March 9, 2020.

^c^ Population density was categorized at the 25th, 50th, 75th, and 90th percentiles, which corresponded to 471, 1022, 1846, and 3951 people per square mile.

**Figure 2.**
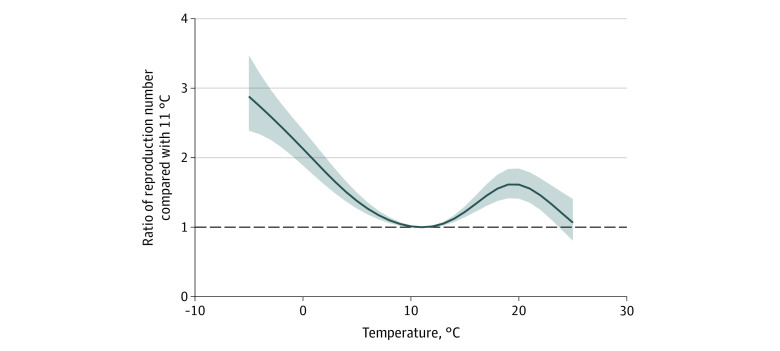
Cumulative Lagged Temperature Dependence of the Instantaneous Reproduction Number of Severe Acute Respiratory Syndrome Coronavirus 2 in 211 US Counties Cumulative exposure-response association between mean daily wet-bulb temperatures and instantaneous reproduction number using a lag period of 4 to 14 days before case identification. The line represents the estimated instantaneous reproduction number at each point along the temperature range compared with 11 °C. The shaded areas represent the 95% CIs. The wet-bulb temperature range for the counties included in the analysis was −9 °C to 25 °C.

The nonlinear association of lagged temperature between 4 and 14 days is reported in Table 2 and Figure 2. Compared with the minimum estimated R_t_ at 11 °C, relative R_t_ increased across the coldest temperatures to a relative R_t_ at 0 °C of 2.13 (95% CI, 1.89-2.40). A smaller peak of the relative R_t_ to 1.61 (95% CI, 1.41-1.84) was estimated at 20 °C, before declining again at higher temperatures. These findings were robust to the addition of 183 less densely populated counties with at least 100 000 residents (eTable and eFigure 3 in the Supplement).

**Figure 3.**
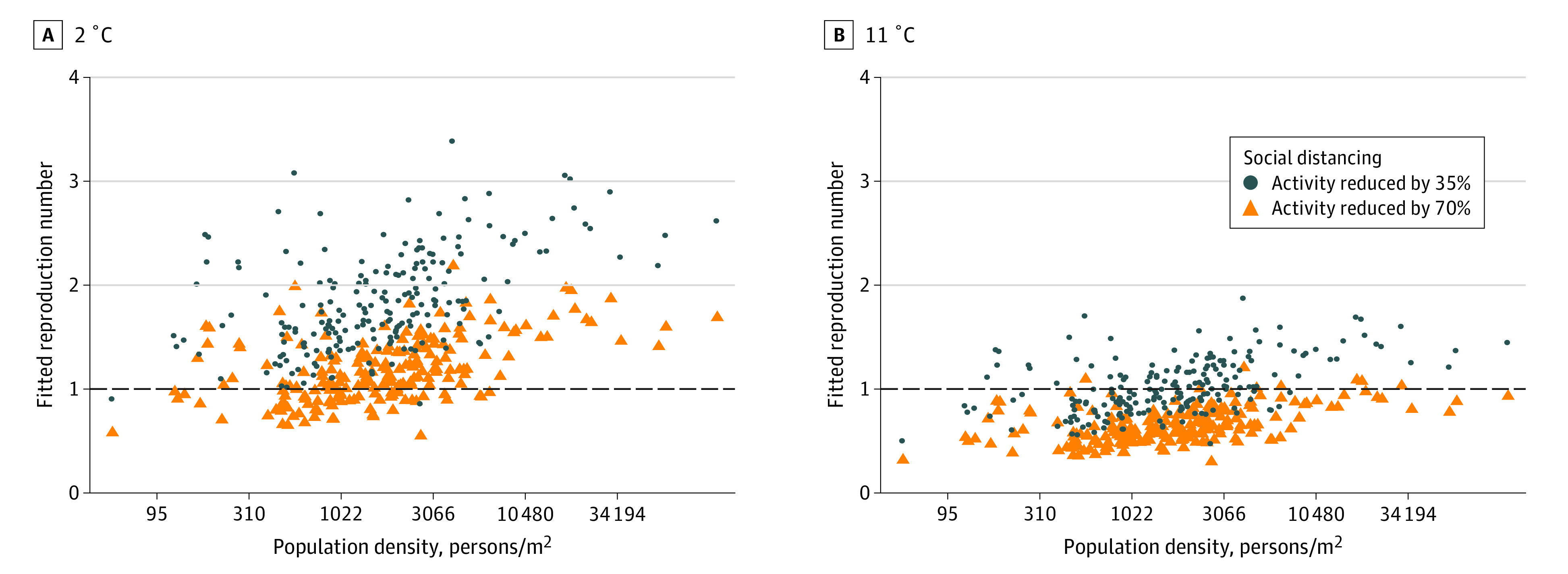
The Association of Social Distancing, Population Density, and Temperature With the Instantaneous Reproduction Number of Severe Acute Respiratory Syndrome Coronavirus 2 in 211 US Counties Each point represents the fitted instantaneous reproduction number of an individual county at 2 °C (A) and 11 °C (B), adjusted for the proportion of residents older than 65 years, with incomes less than 200% of poverty level, and with diabetes. Different levels of social distancing are shown for a 35% reduction and 70% reduction in visits to nonessential businesses.

The standardized association of social distancing, population density, and temperature on R_t_, appears in Figure 3. Assuming social distancing of 35% (ie, halfway between estimates during the US shelter-in-place phase and normal activity), 2 counties (0.9%) were estimated to have a R_t_ less than 1.0 at 2 °C, and 114 counties (54.0%) were estimated to have a R_t_ of less than 1.0 at 11 °C. When visits to nonessential businesses were reduced to the national mean of 70%, the number of counties estimated to have R_t_ less than 1.0 increased to 63 (29.9%) and 202 (95.7%) at 2 °C and 11 °C, respectively. At this 70% reduction in visits to nonessential businesses, 28 and 52 of 53 counties (52.8% and 98.1%, respectively) in the lowest quartile of density achieved this threshold at 2 °C and 11 °C, respectively, compared with 0 and 17 of 21 counties (81.0%) in the top decile at the same temperatures.

No significant interactions were observed for population density and social distancing. Model estimates were stable over time. The overall *R*^2^ of the mixed-effects model was 0.53 based on the covariate associations; the addition of random county and metropolitan area intercepts increased the *R*^2^ to 0.64. Distribution of the in-sample *R*^2^ in a randomly selected 70% of counties over 100 replicates revealed good model fit (eFigure 4 in the Supplement).

## Discussion

In this analysis of 211 US counties, change in social distancing, population density, and wet-bulb daily temperature were associated with the rate of SARS-CoV-2 transmission within a county, as measured by estimated R_t_. Our analysis indicates that of these 3 factors, implementation of social distancing has been the most significant in reducing transmission. In addition, the mitigating association of increased social distancing and moderate increases in wet-bulb daily temperature were most dramatic in counties with higher population density, which had high R_t_ values, consistent with higher R estimates from around the world.^28^

The underlying mechanism for the associations of social distancing and population density on the estimated R_t_ for SARS-CoV-2 is likely associated with increased droplet transmission and potentially airborne transmission when individuals are in closer proximity to each other.^29,30^ However, the association of population density with COVID-19 outcomes may not be limited to transmission. The densest counties also had the highest number of deaths per 100 000 people. The association of more severe disease in higher density areas is hypothesized to be associated with the inoculum effect. The inoculum effect suggests that individuals exposed to a higher viral load at the time of infection will have more severe illness; this is supported by epidemiologic studies for other viruses,^31,32^ in particular for SARS-CoV-1.^33,34,35^ These data support the concept that during a pandemic, people living in highly dense counties are more likely to transmit SARS-CoV-2 and to be exposed to higher inoculums of SARS-CoV-2. This translates not only to more cases of COVID-19 but also to a higher case fatality rate. Data assessing the inoculum effect for SARS-CoV-2 are needed to confirm this hypothesis.

Our analysis, which used well-established methods developed to examine the association of temperature with human health, is also among the first to consider the association of temperature and humidity with SARS-CoV-2 transmission.^22,25^ We found that combined temperature and humidity associations (proxied by wet-bulb temperatures) were nonlinear with the R_t_ and insufficient alone to mitigate R_t_ values below 1 in the absence of considerable social distancing. The nonlinear associations we observed were such that R_t_ ratios decreased, as hypothesized, when wet-bulb temperatures increased to 11 °C. Beyond 11 °C, there was a modest increase in relative R_t_ ratios before the ratios began declining again at higher temperatures.

The nonlinear associations we observed, particularly within the temperate range, are consistent with the inverse relationship of temperature and transmission in animal models for influenza and other coronaviruses.^10,36^ Those studies also found that higher humidity was associated with increased viability and transmission of influenza through fomites, even as aerosol transmission was mitigated with warming temperatures. If SARS-CoV-2 has similar properties to influenza at higher humidity, it could explain our association of wet-bulb temperatures higher than 11 °C with a modest increase in R_t._

Beyond the direct association of temperature and humidity with virus stability, viability, and propagation, it is also possible that changes in temperature and humidity alter human activity. For example, at higher temperatures people may be more likely to congregate in public locations, such as beaches and festivals. Therefore, an increase in R_t_ at higher temperatures could also be explained in part by a decrease in social distancing not measured by our social distancing variable. Regardless of the etiology, we remain cautious in interpreting the association of higher temperature and humidity with R_t_ beyond the temperate range we observed in this analysis. The coming months will allow for additional assessment of R_t_ during more prolonged periods of higher temperature and humidity. These additional observations will help to confirm or refute the attenuated associations at higher temperatures observed in this study.

To date, projections of COVID-19 outcomes have considered large areas, such as countries, provinces, and US states. These models have provided useful information to set expectations for deaths in the United States, to identify potential gaps in health services, such as intensive care unit beds and ventilators, and to guide initial wide-ranging social distancing recommendations from federal and state governments. Our county-level analysis has allowed us to better examine relevant contributions of social distancing, population density, and seasonal weather changes on a given county’s R_t_. This approach gives valuable information on risk of transmission to inform area-specific public policy decisions. It will be important to examine whether the introduction of these associations to models can accurately estimate the likelihood of viral transmission in the future, given that these factors will continue to change. To the degree that predictive modeling efforts are successful, it may demonstrate some validity of using this approach, which incorporates random effects for counties alongside seasonal changes to inform future epidemics.

### Limitations

There are always limitations in observational studies. Generalizability remains a concern, particularly given our focus on larger counties. The 45% of US residents not captured in our analysis were residing in smaller, rural counties, and as such, our models are not applicable to these areas. It is reassuring that the inclusion of an additional 183 more geographically dispersed and less densely populated counties replicated our findings. Second, temperature associations we observed might have been confounded by time period in the analysis, given that outbreaks occurred during spring in parallel with changing weather. However, the addition of time to the model did not appreciably change our results. Third, increases in testing capacity might have biased the models by inflating the total cases reported within each county. It is possible that differences in diagnostic test availability could contribute to the variation detected by the random effects across counties. However, our estimate of R_t_ depended on the rate of change of cases, rather than on the absolute number of cases, and during the period in which this analysis was conducted, test positivity rates, a proxy for testing capacity, were flat.^37^ Furthermore, we smoothed early outbreak case incidences to account for early limited access to diagnostic tests. We intentionally did not include testing capacity as a covariate, so as not to overfit the model (eg, controlling for a factor that was also associated with rising viral transmission itself). Fourth, as the random county and metropolitan area intercepts explained additional variation, there are likely other unmeasured county factors that we did not capture. Our proxy for social distancing used cellular telephone records and may not have captured all movement and gathering within a county; it requires further validation as a proxy for the distancing associations we were measuring. Other unmeasured factors might include commuter automobile traffic, public transportation usage, and domestic and international flights, which had decreased during the study period. It is clear that early local epidemics were seeded by international travel that contributed to early transmission in some locations.^38^ Further investigation will be needed as communities reopen to examine the association of these additional time-varying factors with risk of SARS-CoV-2 transmission.

## Conclusions

The results of this study suggest that social distancing, population density, and daily weather may account for variation in the R_t_ for SARS-COv-2 across the United States. These results may guide policy decisions for managing this pandemic more selectively at the local level throughout the country.

## Notes

Supplement. **eFigure 1. **Average Instantaneous Reproduction Number (R_t_) Superimposed on Scatter of Social Distancing Over Time Across 211 United States Counties **eFigure 2. **Correlation Matrix of Potential Covariates **eFigure 3. **Additional 183 US Counties Included in a Robustness Analysis of Weather and Social Distancing Effects on the Instantaneous Reproduction Number (R_t_) **eFigure 4. **In-Sample *R*^2^ Obtained From 100 Replicates of Random 70% Samples of 211 United States Counties **eTable. **Comparison of Characteristics Between Original 211 and Additional 183 United States Counties Included in a Robustness Analysis of Weather and Social Distancing Effects on the Instantaneous Reproduction Number (R_t_)

## References

[1] Cori A, Ferguson NM, Fraser C, Cauchemez S A new framework and software to estimate time-varying reproduction numbers during epidemics. Am J Epidemiol. 2013;178(9):1505-1512. doi:10.1093/aje/kwt133 24043437 PMC3816335

[2] Fraser C Estimating individual and household reproduction numbers in an emerging epidemic. PLoS One. 2007;2(8):e758-e758. doi:10.1371/journal.pone.0000758 17712406 PMC1950082

[3] Wallinga J, Teunis P Different epidemic curves for severe acute respiratory syndrome reveal similar impacts of control measures. Am J Epidemiol. 2004;160(6):509-516. doi:10.1093/aje/kwh255 15353409 PMC7110200

[4] Children’s Hospital of Philadelphia Exempt research. Accessed May 25, 2020. https://irb.research.chop.edu/exempt-research

[5] von Elm E, Altman DG, Egger M, Pocock SJ, Gøtzsche PC, Vandenbroucke JP; STROBE Initiative The Strengthening the Reporting of Observational Studies in Epidemiology (STROBE) statement: guidelines for reporting observational studies. J Clin Epidemiol. 2008;61(4):344-349. doi:10.1016/j.jclinepi.2007.11.008 18313558

[6] New York Times. COVID-19 data. Updated June 26, 2020. Accessed June 29, 2020. https://github.com/nytimes/covid-19-data

[7] Unacast Social distancing scoreboard. Accessed June 29, 2020. https://www.unacast.com/covid19/social-distancing-scoreboard#methodology

[8] Sheikh A, Sheikh Z, Sheikh A Novel approaches to estimate compliance with lockdown measures in the COVID-19 pandemic. J Glob Health. 2020;10(1):010348. doi:10.7189/jogh.10.010348 32426117 PMC7211415

[9] Li Q, Guan X, Wu P, Early transmission dynamics in Wuhan, China, of novel coronavirus-infected pneumonia. N Engl J Med. 2020;382(13):1199-1207. doi:10.1056/NEJMoa2001316 31995857 PMC7121484

[10] Lowen AC, Mubareka S, Steel J, Palese P Influenza virus transmission is dependent on relative humidity and temperature. PLoS Pathog. 2007;3(10):1470-1476. doi:10.1371/journal.ppat.0030151 17953482 PMC2034399

[11] Shaman J, Kohn M Absolute humidity modulates influenza survival, transmission, and seasonality. Proc Natl Acad Sci U S A. 2009;106(9):3243-3248. doi:10.1073/pnas.0806852106 19204283 PMC2651255

[12] Cheng YT, Lung SC, Hwang JS New approach to identifying proper thresholds for a heat warning system using health risk increments. Environ Res. 2019;170:282-292. doi:10.1016/j.envres.2018.12.059 30599292 PMC7126132

[13] Ross ME, Vicedo-Cabrera AM, Kopp RE, Assessment of the combination of temperature and relative humidity on kidney stone presentations. Environ Res. 2018;162:97-105. doi:10.1016/j.envres.2017.12.020 29289860 PMC5811384

[14] Richardson S, Hirsch JS, Narasimhan M, ; and the Northwell COVID-19 Research Consortium Presenting characteristics, comorbidities, and outcomes among 5700 patients hospitalized with COVID-19 in the New York City area. JAMA. 2020;323(20):2052-2059. doi:10.1001/jama.2020.6775 32320003 PMC7177629

[15] NAICS Association Six-digit NAICS codes and titles. Accessed March 29, 2020. https://www.naics.com/six-digit-naics/

[16] Social Explorer Data dictionary: ACS 2018 (5-year estimates). Accessed March 29, 2020. https://www.socialexplorer.com/data/ACS2018_5yr/metadata/?ds=SE&table

[17] US Census Bureau Commuting flows. Accessed June 29, 2020. https://www.census.gov/topics/employment/commuting/guidance/flows.html

[18] US Centers for Disease Control and Prevention 500 cities: local data for better health. Accessed March 25, 2020. https://www.cdc.gov/500cities/

[19] Pan A, Liu L, Wang C, Association of public health interventions with the epidemiology of the COVID-19 outbreak in Wuhan, China. JAMA. 2020;323(19):1915-1923. doi:10.1001/jama.2020.6130 32275295 PMC7149375

[20] Nadaraya EA On Estimating Regression. Theory Probability Applications. 1964;9(1):141-142. doi:10.1137/1109020

[21] Bierens HJ The Nadaraya–Watson kernel regression function estimator In: Topics in Advanced Econometrics. New York: Cambridge University Press; 1994:212-247. doi:10.1017/CBO9780511599279.011

[22] Gasparrini A, Armstrong B, Kenward MG Distributed lag non-linear models. Stat Med. 2010;29(21):2224-2234. doi:10.1002/sim.3940 20812303 PMC2998707

[23] Armstrong B Models for the relationship between ambient temperature and daily mortality. Epidemiology. 2006;17(6):624-631. doi:10.1097/01.ede.0000239732.50999.8f 17028505

[24] Tasian GE, Pulido JE, Gasparrini A, ; Urologic Diseases in America Project Daily mean temperature and clinical kidney stone presentation in five U.S. metropolitan areas: a time-series analysis. Environ Health Perspect. 2014;122(10):1081-1087. doi:10.1289/ehp.1307703 25009122 PMC4181925

[25] Gasparrini A, Guo Y, Hashizume M, Mortality risk attributable to high and low ambient temperature: a multicountry observational study. Lancet. 2015;386(9991):369-375. doi:10.1016/S0140-6736(14)62114-0 26003380 PMC4521077

[26] Vicedo-Cabrera AM, Goldfarb DS, Kopp RE, Song L, Tasian GE Sex differences in the temperature dependence of kidney stone presentations: a population-based aggregated case-crossover study. Urolithiasis. 2020;48(1):37-46. doi:10.1007/s00240-019-01129-x 30900001 PMC7357996

[27] Gasparrini A Distributed lag linear and non-linear models in R: the package dlnm. J Stat Softw. 2011;43(8):1-20. doi:10.18637/jss.v043.i08 22003319 PMC3191524

[28] Sanche S, Lin YT, Xu C, Romero-Severson E, Hengartner N, Ke R High contagiousness and rapid spread of severe acute respiratory syndrome coronavirus 2. Emerg Infect Dis. 2020;26(7):1470-1477. doi:10.3201/eid2607.200282 32255761 PMC7323562

[29] van Doremalen N, Bushmaker T, Morris DH, Aerosol and surface stability of SARS-CoV-2 as compared with SARS-CoV-1. N Engl J Med. 2020;382(16):1564-1567. doi:10.1056/NEJMc2004973 32182409 PMC7121658

[30] Bahl P, Doolan C, de Silva C, Chughtai AA, Bourouiba L, MacIntyre CR Airborne or droplet precautions for health workers treating coronavirus disease 2019? J Infect Dis. Published April 16, 2020. doi:10.1093/infdis/jiaa189 PMC7184471 32301491

[31] Poulsen A, Qureshi K, Lisse I, A household study of chickenpox in Guinea-Bissau: intensity of exposure is a determinant of severity. J Infect. 2002;45(4):237-242. doi:10.1053/jinf.2002.1049 12423611

[32] Paulo AC, Correia-Neves M, Domingos T, Murta AG, Pedrosa J Influenza infectious dose may explain the high mortality of the second and third wave of 1918-1919 influenza pandemic. PLoS One. 2010;5(7):e11655-e11655. doi:10.1371/journal.pone.0011655 20668679 PMC2909907

[33] Chu C-M, Poon LLM, Cheng VCC, Initial viral load and the outcomes of SARS. CMAJ. 2004;171(11):1349-1352. 15557587 10.1503/cmaj.1040398 PMC527336

[34] Virlogeux V, Fang VJ, Wu JT, Brief report: incubation period duration and severity of clinical disease following severe acute respiratory syndrome coronavirus infection. Epidemiology. 2015;26(5):666-669. doi:10.1097/EDE.0000000000000339 26133021 PMC4889459

[35] Watanabe T, Bartrand TA, Weir MH, Omura T, Haas CN Development of a dose-response model for SARS coronavirus. Risk Anal. 2010;30(7):1129-1138. doi:10.1111/j.1539-6924.2010.01427.x 20497390 PMC7169223

[36] Ijaz MK, Brunner AH, Sattar SA, Nair RC, Johnson-Lussenburg CM Survival characteristics of airborne human coronavirus 229E. J Gen Virol. 1985;66(Pt 12):2743-2748. doi:10.1099/0022-1317-66-12-2743 2999318

[37] Children’s Hospital of Philadelphia COVID-lab mapping COVID-19 in your community. Accessed May 15, 2020. https://policylab.chop.edu/covid-lab-mapping-covid-19-your-community

[38] Wells CR, Sah P, Moghadas SM, Impact of international travel and border control measures on the global spread of the novel 2019 coronavirus outbreak. Proc Natl Acad Sci U S A. 2020;117(13):7504-7509. doi:10.1073/pnas.2002616117 32170017 PMC7132249

