# Supplementary material for: Association of Social Distancing, Population Density, and Temperature With the Instantaneous Reproduction Number of SARS-CoV-2 in Counties Across the United States
Source: JAMA Netw Open. 2020 Jul 23;3(7):e2016099. doi: 10.1001/jamanetworkopen.2020.16099 (PMC7378754; doi:10.1001/jamanetworkopen.2020.16099)
Supplement: Supplement. — eFigure 1. Average Instantaneous Reproduction Number (Rt) Superimposed on Scatter of Social Distancing Over Time Across 211 United States Counties eFigure 2. Correlation Matrix of Potential Covariates eFigure 3. Additional 183 US Counties Included in a Robustness Analysis of Weather and Social Distancing Effects on the Instantaneous Reproduction Number (Rt) eFigure 4. In-Sample R2 Obtained From 100 Replicates of Random 70% Samples of 211 United States Counties eTable. Comparison of Characteristics Between Original 211 and Additional 183 United States Counties Included in a Robustness Analysis of Weather and Social Distancing Effects on the Instantaneous Reproduction Number (Rt) [file jamanetwopen-3-e2016099-s001.pdf]

Supplementary Online Content

Rubin D, Huang J, Fisher BT, et al. Association of social distancing, population density, and temperature with the instantaneous reproduction number of SARS-CoV-2 in counties across the United States. *JAMA Netw Open*. 2020;3(7):e2016099. doi:10.1001/jamanetworkopen.2020.16099

eFigure 1. Average Instantaneous Reproduction Number (R_t) Superimposed on Scatter of Social Distancing Over Time Across 211 United States Counties

eFigure 2. Correlation Matrix of Potential Covariates

eFigure 3. Additional 183 US Counties Included in a Robustness Analysis of Weather and Social Distancing Effects on the Instantaneous Reproduction Number (R_t)

eFigure 4. In-Sample R^2 Obtained From 100 Replicates of Random 70% Samples of 211 United States Counties

eTable. Comparison of Characteristics Between Original 211 and Additional 183 United States Counties Included in a Robustness Analysis of Weather and Social Distancing Effects on the Instantaneous Reproduction Number (R_t)

This supplementary material has been provided by the authors to give readers additional information about their work.

© 2020 Rubin D et al. *JAMA Network Open.*

eFigure 1: Average Instantaneous Reproduction Number (R_t) Superimposed on Scatter of Social Distancing Over Time Across 211 United States Counties

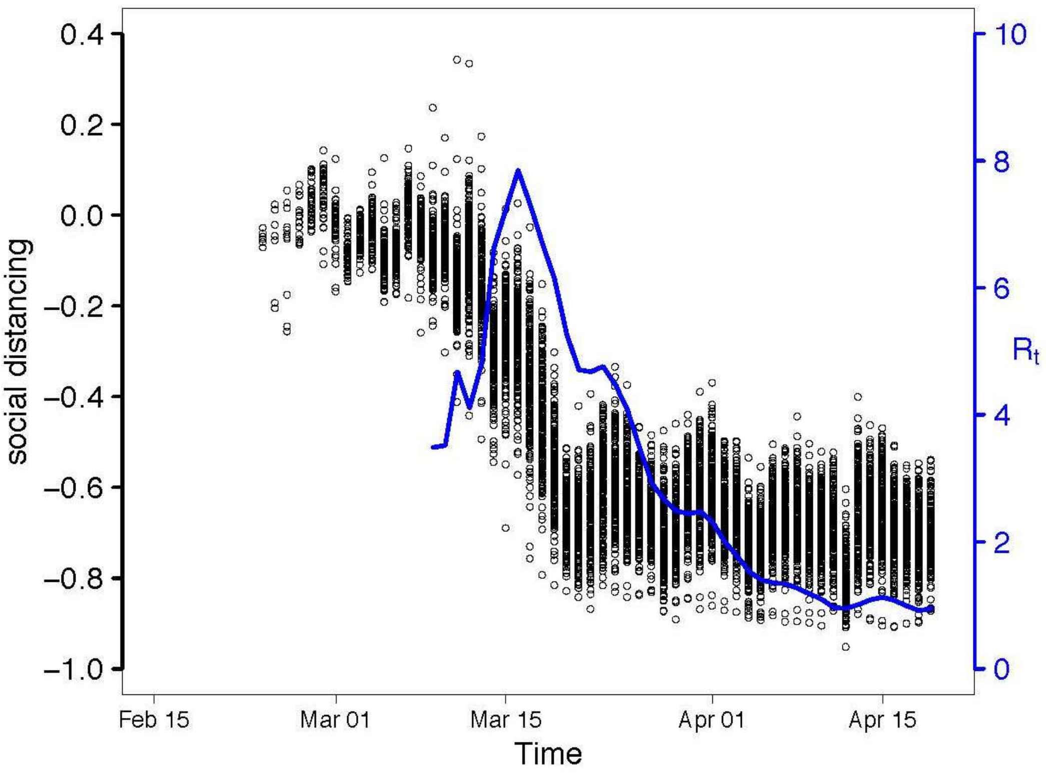

eFigure 1. Instantaneous reproduction number (R_t) estimated from the methods of Wallinga and Teunis (2004). Social distancing measured as the proportional change in in visits to non-essential businesses.

© 2020 Rubin D et al. *JAMA Network Open.*

eFigure 2. Correlation Matrix of Potential Covariates

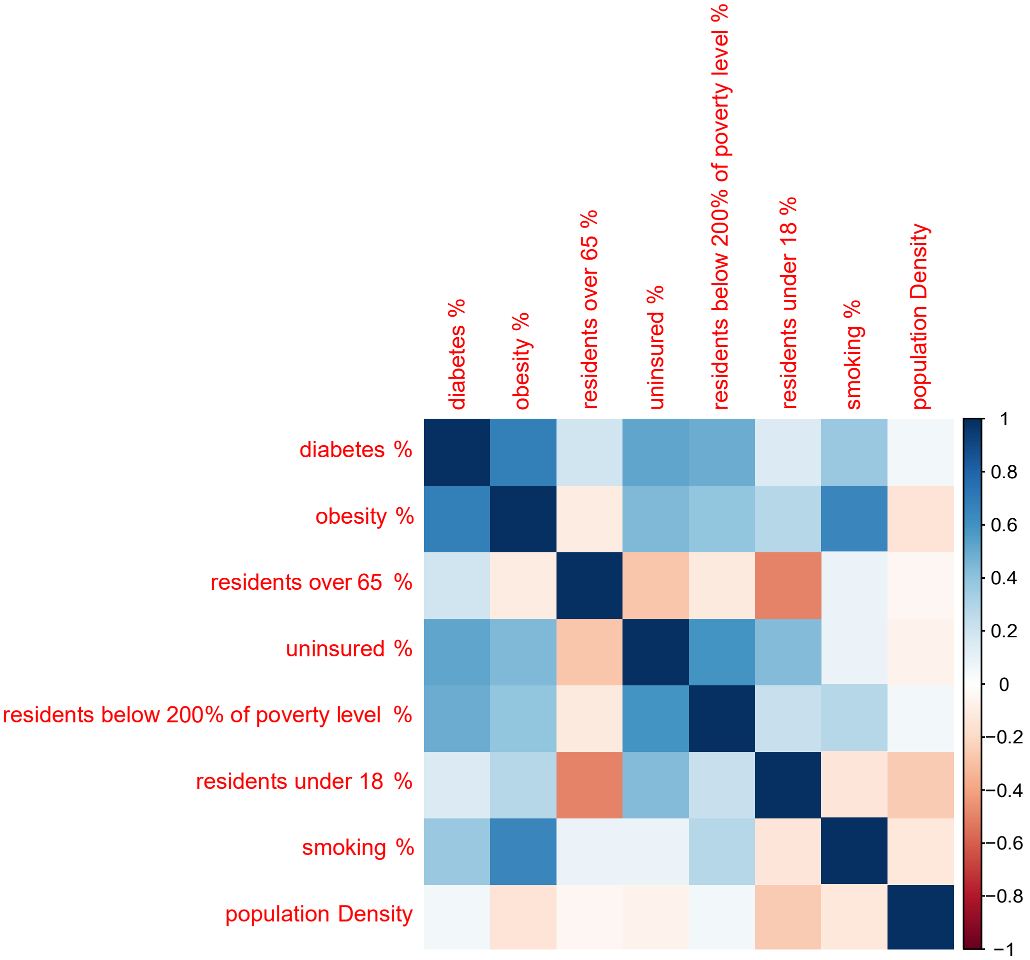

eFigure 2. Each block represents magnitude of Pearson correlation between pairs of covariates. Darker colors indicate higher correlation.

© 2020 Rubin D et al. *JAMA Network Open.*

eFigure 3. Additional 183 US Counties Included in a Robustness Analysis of Weather and Social Distancing Effects on the Instantaneous Reproduction Number (Rt)

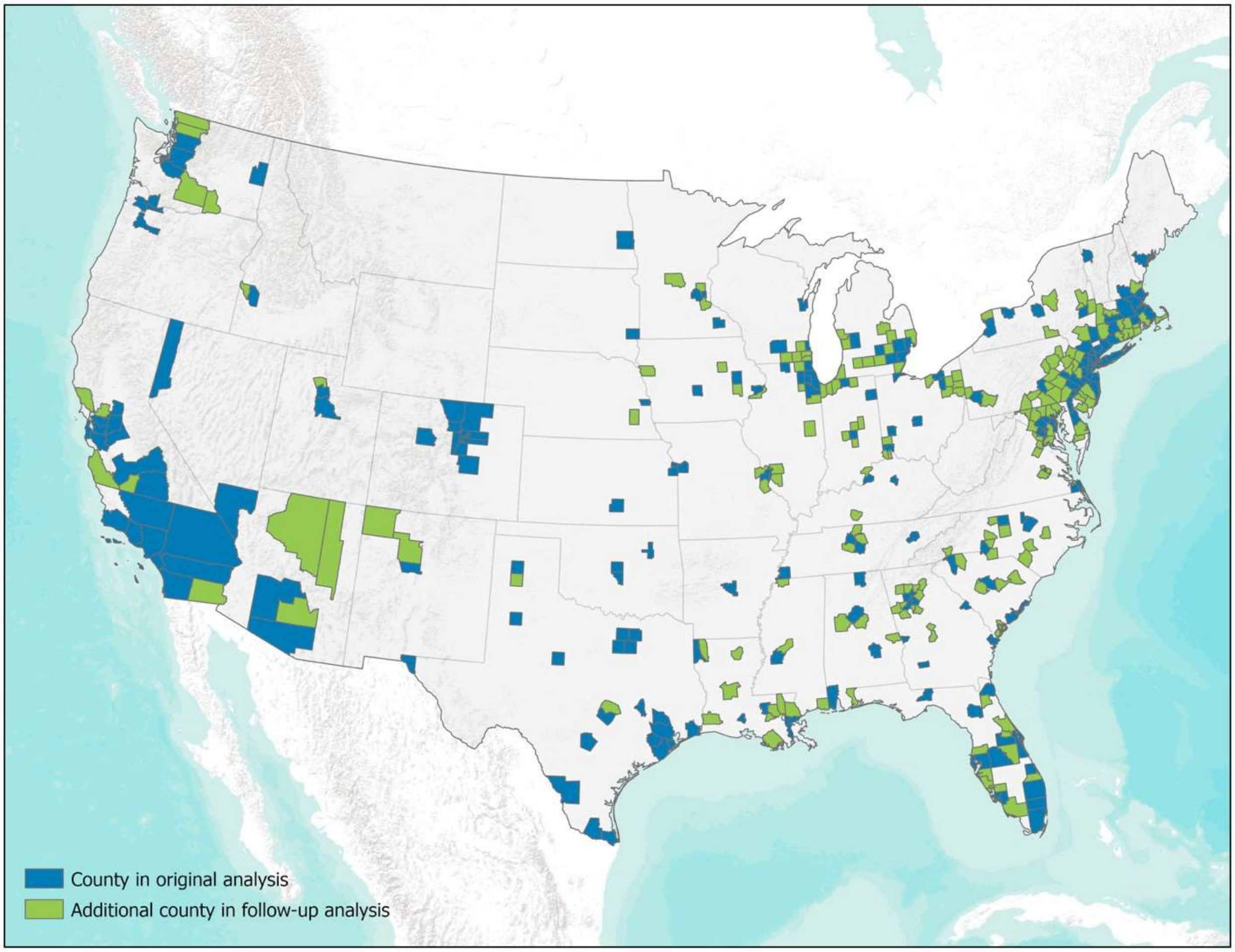

eFigure 4. Locations of counties in original analysis (blue) and those added to the follow-up analysis (green).

© 2020 Rubin D et al. *JAMA Network Open.*

eFigure 4: In-Sample R^2 Obtained From 100 Replicates of Random 70% Samples of 211 United States Counties

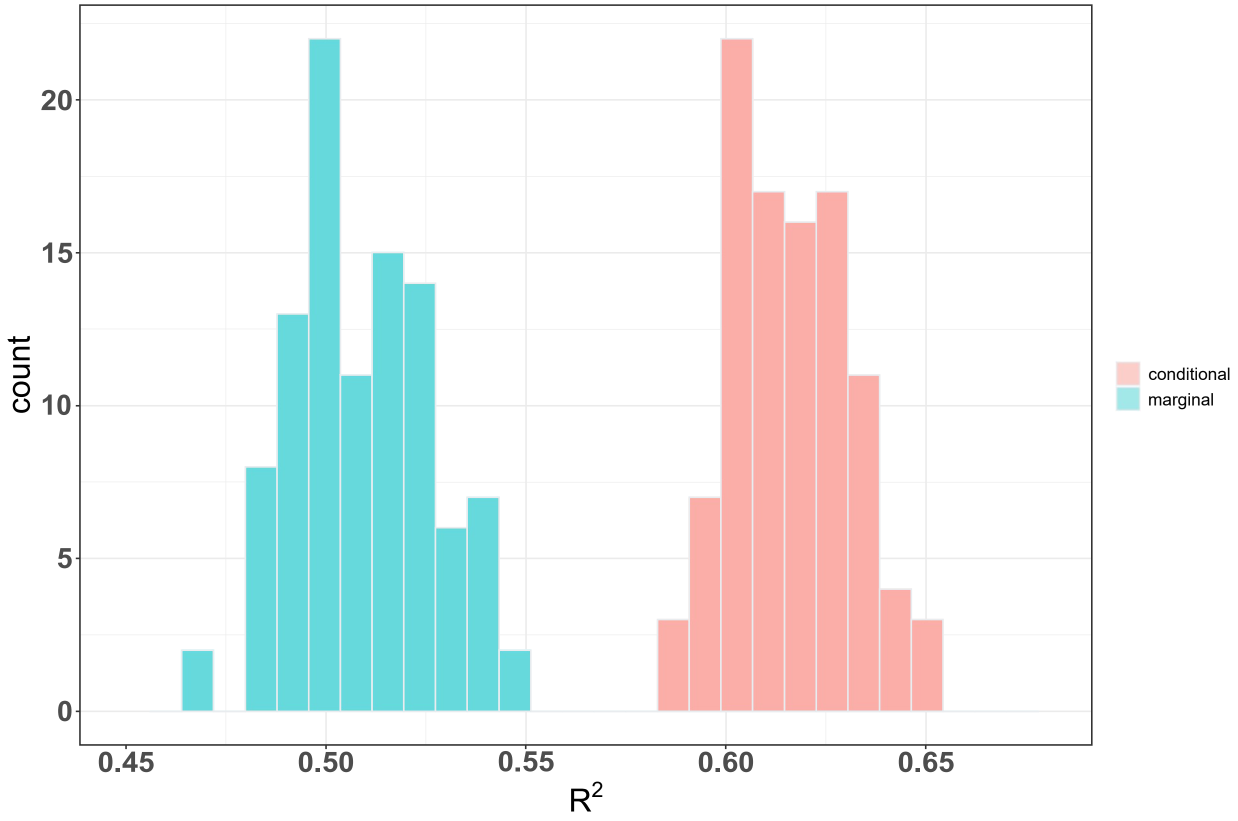

eFigure 4. R-squared obtained from mixed effects model described in the methods of the manuscript.

© 2020 Rubin D et al. *JAMA Network Open.*

eTable. Comparison of Characteristics Between Original 211 and Additional 183 United States Counties Included in a Robustness Analysis of Weather and Social Distancing Effects on the Instantaneous Reproduction Number (R_t)

**Characteristics**	Original 211 Counties	Additional 183 Counties
Population density median (IQR), per mi^2	1,022.7 (471.2 - 1,846.0)	343.4 (225.4 - 534.3)
% Northeast	22.7	27.9
% Midwest	19.4	25.7
% South	36.5	37.7
% West	21.3	8.6
Hypertension, mean (SD), %	30.7 (4.2)	32.5 (3.7)
Diabetes, mean (SD), %	9.9 (1.8)	10.1 (1.4)
Regular smokers, mean (SD), %[b]	16.5 (2.5)	18.3 (2.4)
BMI > 30, mean (SD), %	30.1 (3.9)	31.6 (2.7)
Age <18 years, median (IQR), %	22.7 (21.4 - 24.5)	23.0 (20.7 - 24.7)
Age 18 to 34 years, median (IQR), %	24.1 (22.3 - 26.2)	21.5 (20.2 - 23.1)
Age 35 to 64 years, median (IQR), %	38.4 (36.9 - 40.1)	39.6 (37.4 - 40.9)
Age ≥ 65 years, median (IQR), %	13.8 (12.4 - 15.4)	15.5 (13.3 - 17.2)

© 2020 Rubin D et al. *JAMA Network Open.*

Low Income, mean (SD), %[c]	30.6 (9.3)	29.3 (8.9)
Uninsured, mean (SD), %	9.1 (4.6)	7.9 (3.5)
Change in visits to non-essential businesses **2/24/20-3/8/20,** mean (SD), %[d]	-2.7 (6.3)	-2.3 (6.6)
Change in visits to non-essential businesses **4/6/20-4/19/20,** mean (SD), %[d]	-68.7 (-7.9)	-61.4 (9.3)
Change in visits to non-essential businesses **5/28/20-6/11/20,** mean (SD), %[d]	-35.7 (16.6)	-20.8 (18.9)
Daily wet bulb temperature, median (IQR), °C[e]	13.1 (5.7 - 18.8)	12.6 (5.0 - 18.9)
Cases per 100,000 people on 6/12/20, median (IQR)	518.8 (277.1 - 1,110.5)	508.8 (339.1 - 783.8)
Deaths per 100,000 people on 6/12/20, median (IQR)	20.5 (9.4 - 53.7)	23.9 (12.2 - 42.0)
R in the first two weeks, mean (SD)	3.6 (3.0)	2.6 (2.1)

Abbreviations: SD, standard deviation; IQR, interquartile range; BMI, body mass index (calculated as weight in kilograms divided by height in meters squared);
[a] All characteristics were obtained from the American Survey (2018) except health data, which were obtained from the CDC Behavioral Risk Factor Surveillance System (2017), social distancing, obtained from Unacast (2020), and wet bulb temperature, from NOAA (2020).
[b] Regular smokers was defined as adult respondents who reported smoking ≥100 in their life and currently smoke at least some days.

[c] Low income was defined as <200% poverty level
[d] Visits to non-essential businesses obtained from Uncast, change from the average non-essential business visits during matching days of the week before 3/9/2020.
[e] Daily wet-bulb temperatures from 2/1/2020 - 6/12/2020 were calculated by averaging the hourly recordings from weather stations that contribute to the National Oceanic and Atmospheric Administration's Local Climatological Data

© 2020 Rubin D et al. *JAMA Network Open.*
